# Transgenic Chickens Expressing the 3D8 Single Chain Variable Fragment Protein Suppress Avian Influenza Transmission

**DOI:** 10.1038/s41598-017-05270-8

**Published:** 2017-07-19

**Authors:** Sung June Byun, Seong-su Yuk, Ye-Jin Jang, Hoonsung Choi, Mi-Hyang Jeon, TO Erdene-Ochir, Jung-Hoon Kwon, Jin-Yong Noh, Jeom Sun Kim, Jae Gyu YOO, Chang-Seon Song

**Affiliations:** 1 Animal Biotechnology Division, National Institute of Animal Science, Rural Development Administration, Suwon, 441-706 Republic of Korea; 2 Department of Avian Disease Laboratory, College of Veterinary Medicine, Konkuk University, Seoul, Korea

## Abstract

The 3D8 single chain variable fragment (scFv) is a mini-antibody that causes unusual sequence-independent nuclease activity against all types of nucleic acids. We used recombinant lentiviruses to generate transgenic chickens expressing the 3D8 scFv gene under the control of the chicken β-actin promoter. From 420 injected embryos, 200 chicks (G0) hatched and were screened for the 3D8 scFv using PCR, and 15 chicks were identified as transgenic birds expressing the transgene in their semen. The G0 founder birds were mated with wild-type hens to produce seven transgenic chicks (G1). 3D8 scFv expression in the chicken embryonic fibroblasts (CEFs) was verified by RT-PCR and Western blot analysis. Immunofluorescence staining for 3D8 scFv in the CEFs revealed that the 3D8 scFv protein was primarily cytosolic. To identify 3D8 scFv anti-viral activity, wild-type and two transgenic CEF lines were infected with H9N2 avian influenza virus (AIV). We selected one line of transgenic chickens that exhibited the lowest number of plaque-forming units to be challenged with H9N2 virus. The challenge experiment revealed that contact exposed transgenic chickens expressing 3D8 scFv exhibited suppressed viral shedding. This results suggest that the transgenic chickens developed in this study could be useful for controlling potential within-flock AIV transmission.

## Introduction

Avian influenza (AI) belong to type A influenza virus have potential to infect a wide range of animal species, and its transmission characteristics make it a global threat to domestic birds as well as mammals, including humans^1^. In the poultry industry, when diseases are endemic and/or biosecurity measures are impractical to enforce, vaccination is one of the most widely used tools for the control of AI infection^2^; however, the diversity of viral antigenic subtypes and their potential for evolutionary shifts and genetic drift are major hurdles that inhibit the achievement of sterile immunity by vaccination, even for antigenically well-matched viruses^3, 4^. Therefore, novel approaches for controlling AI infections are needed, and alternative strategies for the prevention and control of influenza virus infection are being developed.

To date, several antiviral research approaches have been utilized to control AI. Inhibitors such as antiviral drugs act to block the activity of viral proteins involved in the targeted virus’ entry into, replication within or departure from host cells^5, 6^; however, there is concern that antiviral drug abuse in farm animals could lead to the growth of antiviral-resistant mutant viruses. Monoclonal antibody targeting of viral proteins has had limited success because of the difficulty in identifying suitable epitopes for neutralization as well as viral antigen shift and drift^7^. Another antiviral approach operates by degrading the viral RNA genome using siRNAs, which are sequences that target avian influenza virus (AIV) ribonucleoprotein or nucleoprotein genes^8, 9^. In a more practical approach, recombinant proteins, such as cytokines and probiotics, have been utilized as feed additives to improve immunity against AI^10–13^. Notably, all of the antiviral approaches described above are limited to a degree because of their high cost and difficulty of application *in vivo*.

Ribonucleases (RNases) represent another antiviral therapy approach to degrading viral RNA genomes. Earlier studies demonstrated that RNases originating from different hosts exhibit effective antiviral activities towards the human immunodeficiency virus (HIV-1) and potato spindle tuber viroid (PSTVd), although the antiviral effects of these RNases depends on their RNA-hydrolyzing activity^14–16^. The 3D8 single chain variable fragment (3D8 scFv) is produced from the variable domains of heavy (VH) and light (VL) DNA products of an anti-DNA monoclonal antibody isolated from the spleen cells of the MRL-lpr/lpr mouse, which spontaneously develops an autoimmune syndrome resembling human systemic lupus erythematosus^17^. 3D8 scFv is distinctive in that the recombinant protein can internalize via caveolae/lipid raft pathway-mediated endocytosis via initial interactions with cell-surface heparin sulfate proteoglycans and then accumulate in the cytosol without being translocated to the nucleus^18^. Moreover, a recent study demonstrated that 3D8 scFv exhibits hydrolytic activity towards RNA and DNA viruses^19, 20^. These study demonstrated that the 3D8 scFv gene may have the potential for use as an effective new antiviral material that can prevent and control AIV by hydrolyzing the AIV genome.

We investigated genetic modifications as an approach for controlling AI in commercial poultry by introducing a 3D8 scFv gene customized to have an increased affinity for AI nucleoproteins. Here, we generated a line of transgenic chickens expressing the 3D8 scFv gene, evaluated transgenic 3D8 scFv expression and performed challenge experiments using H9N2 AIV.

## Results

### Production of transgenic chickens expressing 3D8 scFv

**Figure 1:**
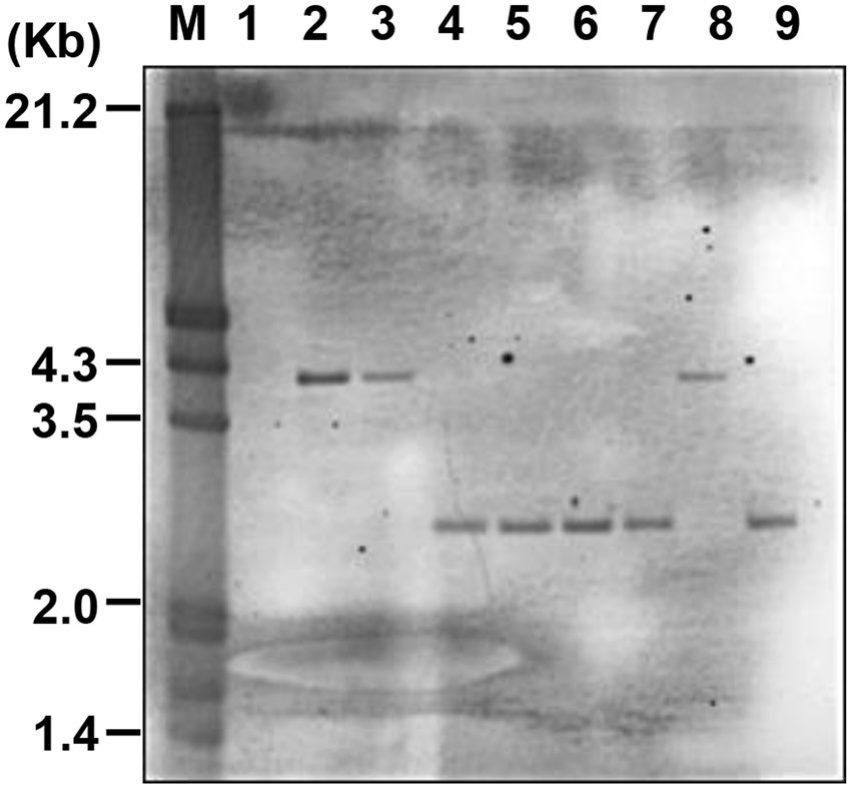
Southern blot analysis of the transgenic chickens. Genomic DNA samples were extracted from whole blood samples from wild-type and transgenic chickens (G1 and G2), digested with EcoRV, electrophoresed, blotted, and hybridized with a probe for the 3D8 scFv gene. M, DIG-labeled DNA marker; lane 1, wild-type chicken; lane 2, P-54; lane 3, P-54 offspring; lane 4, N-82; lane 5, N-95; lane 6, N-165; lane 7, N-183; lane 8, P-196; lane 9, N-238.

**Figure 2:**
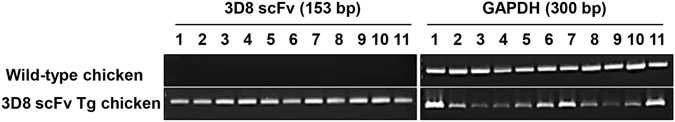
RT-PCR analysis of 3D8 scFv mRNA in transgenic and wild-type chicken tissues. 3D8 scFv mRNA is present in the trachea (1), ovary/testis (2), kidney (3), spleen (4), pancreas (5), brain (6), lung (7), liver (8), heart (9), muscle (10), and intestines (11).

The CBA-3D8 scFv-HA-IRES-puro lentiviral vector (Supplementary Figure S1) was pseudotyped with vesicular stomatitis virus glycoprotein (VSV-G) and used to produce transgenic birds. A total of 420 embryos (stage-x) were injected with approximately 100,000 virus particles each, and 200 G0 chicks (47.6%) ultimately hatched. To screen for the transgenic chickens, 185 chicks were raised to sexual maturation, and semen samples from 92 surviving roosters were screened by PCR. Fifteen cockerels (G0) were identified as possessing the 3D8 scFv gene presented in the germ line (data not shown). Three transgenic roosters (J-21, J-168, and J-186) were randomly selected and crossed to wild-type counterparts, and 1302 chicks (J-21, 459; J-168, 335; J-186, 508) were produced and screened. From these chicks, 9 transgenic G1 offspring were identified by PCR (data not shown). The J-168 rooster did not produce any transgenic progeny, whereas the J-21 and J-186 roosters produced 6 and 3 progeny, respectively. During G1 transgenic chick growth, two died (N-140 and P-296) suddenly of unknown causes. A Southern blot analysis was used to verify the presence of the 3D8 scFv insertion in seven G1 birds (N-82, N-95, N-165, N-183, N-238, P-54, and P-196) and one G2 bird (offspring of P-54) (Fig. 1). The transgenic chickens were divided in two groups according to their gene insertion positions, which resulted from the different founder transgenic chickens (J-21 and J-186). Because the G1 transgenic birds consisted of one male and six females, the transgenic G1 animals were crossbred to wild-type hens or roosters to generate G2 transgenic chickens. The 187 resulting G2 progeny were screened by PCR to assess the presence of the transgene (data not shown). The ratio of transgenic to non-transgenic offspring (91/187, 49%) did not significantly differ from the expected Mendelian ratio, and the results from the G1 generation of transgenic chickens are summarized in Supplementary Table S2. The expression of 3D8 scFv in the transgenic and wild-type chicken tissues was analyzed by RT-PCR using the 3D8 scFv-specific primer. In the transgenic chickens, the transgene was expressed in all tissues, whereas in the wide-type chickens, 3D8 scFv was not expressed in any tissues (Fig. 2). We also investigated behavioral pattern and weight gain kinetics in case of possible phenotypic disorder derived from the production. The transgenic chickens showed normal behavioral properties in hearing, seeing, vocalizing, smelling, feeding and even mating. We compared relative weight gains of the transgenic chicken with that of other lines transgenic or non-transgenic chickens (Supplementary Figure S4). Accordingly, we presumed no significant in phenotyping relationship between 3D8 protein and lentiviral vector-derived factor.

### 3D8 scFv expression in transgenic CEFs

**Figure 3:**
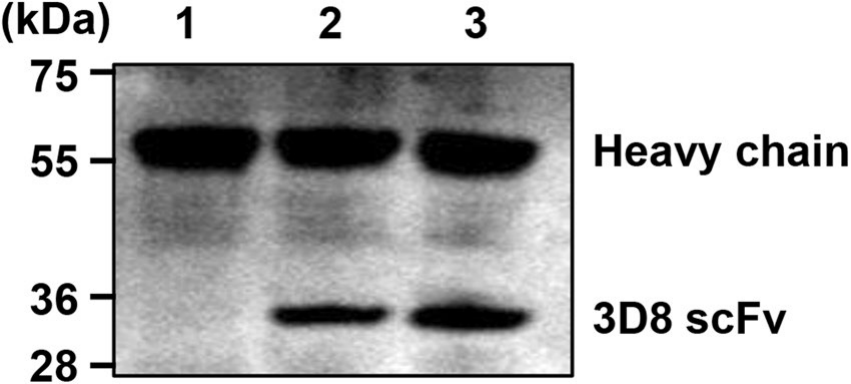
Western blot analysis of 3D8 scFv proteins in transgenic and non-transgenic CEFs. The 3D8 scFv gene was expressed in transgenic CEFs. A heavy chain was also detected in all samples because of the immunoprecipitation. M: standard marker protein sizes; lane 1, wild-type CEF; lane 2, transgenic CEF (E-185); lane 3, transgenic CEF (D-280).

**Figure 4:**
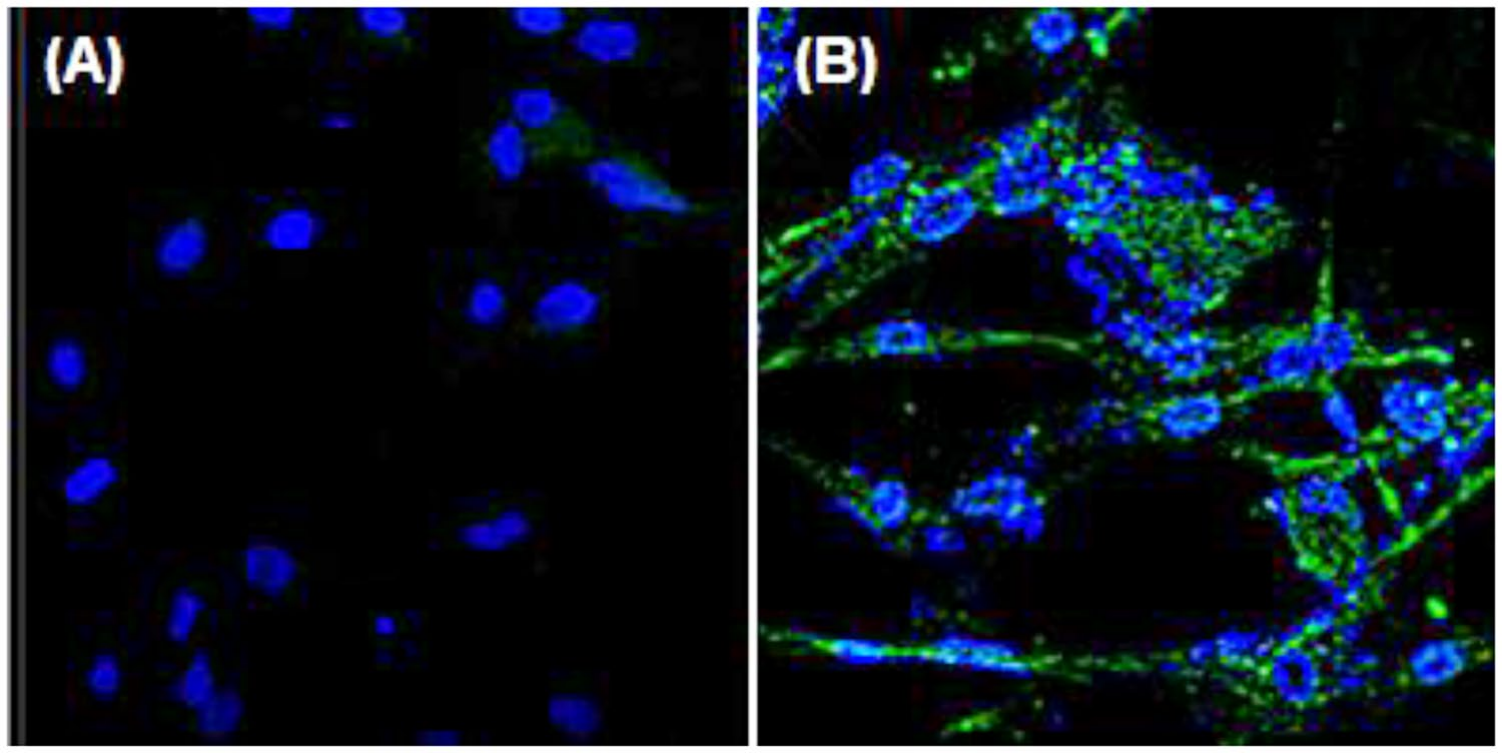
Cells were stained with rabbit anti-HA tag antibody and secondary FITC-anti-rabbit antibody for confocal microscopy; DAPI-stained nuclei are colored blue. Wild-type (**A**), transgenic CEFs (**B**).

To characterize the expression and distribution of 3D8 scFv protein within the transgenic CEFs, Western blotting and confocal microscopy analyses were performed. Because the Western blot analysis did not show any positive bands under normal conditions, we performed an immunoprecipitation and observed a band at approximately 30 kDa, which corresponded to the 3D8 scFv protein (Fig. 3). 3D8 scFv appeared to be expressed at low levels. Similarly, confocal microscopy revealed a weak cytoplasmic fluorescence signal (Fig. 4). Together, these data indicate that transgenic CEFs expressed 3D8 scFv at low levels. To localize the inserted 3D8 scFv gene in transgenic chicken, we traced the sequence and identified that the vector was inserted into the intron sequence between exon 18 and 19 of ABCA4 transcript variant 1 (Supplementary Figure S3).

### Reduced viral growth in transgenic CEFs

**Figure 5:**
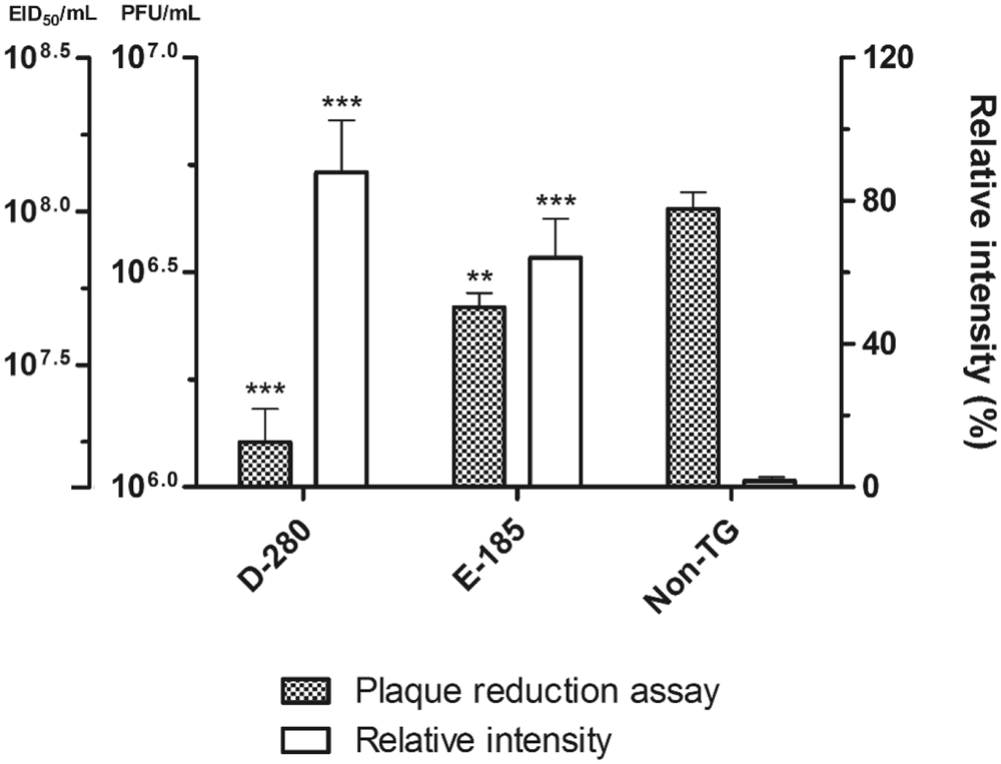
Plaque reduction assay from 3D8 scFv-expressing H9N2-infected CEFs with intensity of the Western blot bands. MDCK monolayers were infected with either undiluted or 10-fold diluted supernatant from 3D8 scFv-expressing H9N2-infected CEFs and overlaid with agar. After 48 hours, visible plaques were detected by crystal violet staining and then counted. The data are shown as the mean number of plaque-forming units for each assay in triplicate, and extrapolated 50% egg infectious dose were noted. Western blot band intensities of 3D8 scFv-expressing CEFs were quantified using Image J 1.31 V software. Statistical significance: **p < 0.01; ***p < 0.001 by ANOVA with Tukey-Kramer post-test when compared with non-TG group.

To assess the antiviral activities of the 3D8 scFv protein in CEFs, we compared the viral growth in transgenic and non-transgenic CEFs using a plaque reduction assay. Compared with the non-transgenic CEFs, the transgenic CEF supernatant exhibited significantly reduced plaque formation in MDCK cells. Compared with the plaque formation results for the non-transgenic CEFs, reductions of 71.1% and 41.1% were observed for the 10-fold diluted D-280 and E-185 CEF plaques, respectively (Fig. 5). Meanwhile, relative intensity of Western blot bands in Fig. 3 were 89.2% (D-280) and 65.1% (E-185). These results indicate that H9N2 virus growth was inhibited in transgenic CEFs as relative expression of 3D8 scFv increase, which results in reduced plaque formation on MDCK cells. In addition, we performed real-time PCR, assessing the expression level of the 3D8 scFv gene in the bronchi of wild-type and transgenic chickens, The 3D8 scFv was highly expressed in the transgenic chicken (D-280) (Supplementary Figure S5). Although the 3D8 scFv expression level in the bronchi was not enough to reduce viral shedding from direct H9N2 infection through nasal inoculation, it could suppress the viral shedding in the contact-exposed chickens as shown in the animal experiment.

### Reduced viral shedding of transgenic chickens after contact transmission of AIV

**Figure 6:**
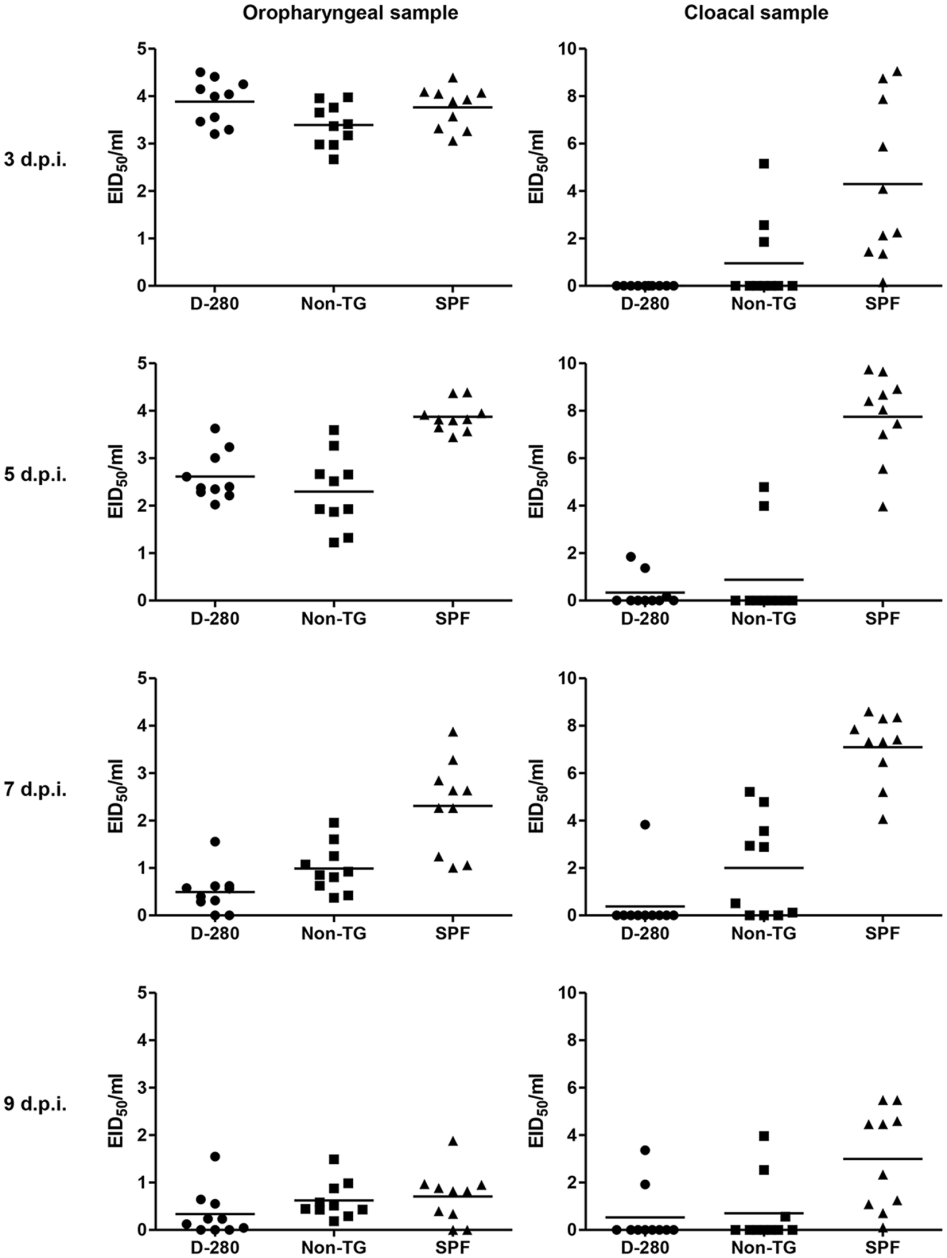
Viral shedding of directly challenged transgenic chickens. Twenty three-week-old non-transgenic chickens (non-TG) and transgenic chickens (D-280) were housed each containment cages in a BSL2 animal facility, and an additional twenty three-week-old SPF chickens were housed as a control group. Ten chickens from each group were intranasally challenged with 100 ${\rm{\mu }}{\rm{l}}$ of 10^7.5^ EID_50_/mL H9N2 virus. The other 10 naïve chickens in each group were co-housed in the same containment isolator from 6 hour post-inoculation. Oropharyngeal and cloacal swab samples were collected at 3, 5, 7, and 9 days post-inoculation (dpi) from the challenged chickens and the viral RNA was quantified by real-time RT-PCR. Each cycle threshold value was extrapolated to egg infectious dose fifty and mean viral shedding titers were calculated. ***p < 0.001 by unpaired *t*-test.

**Figure 7:**
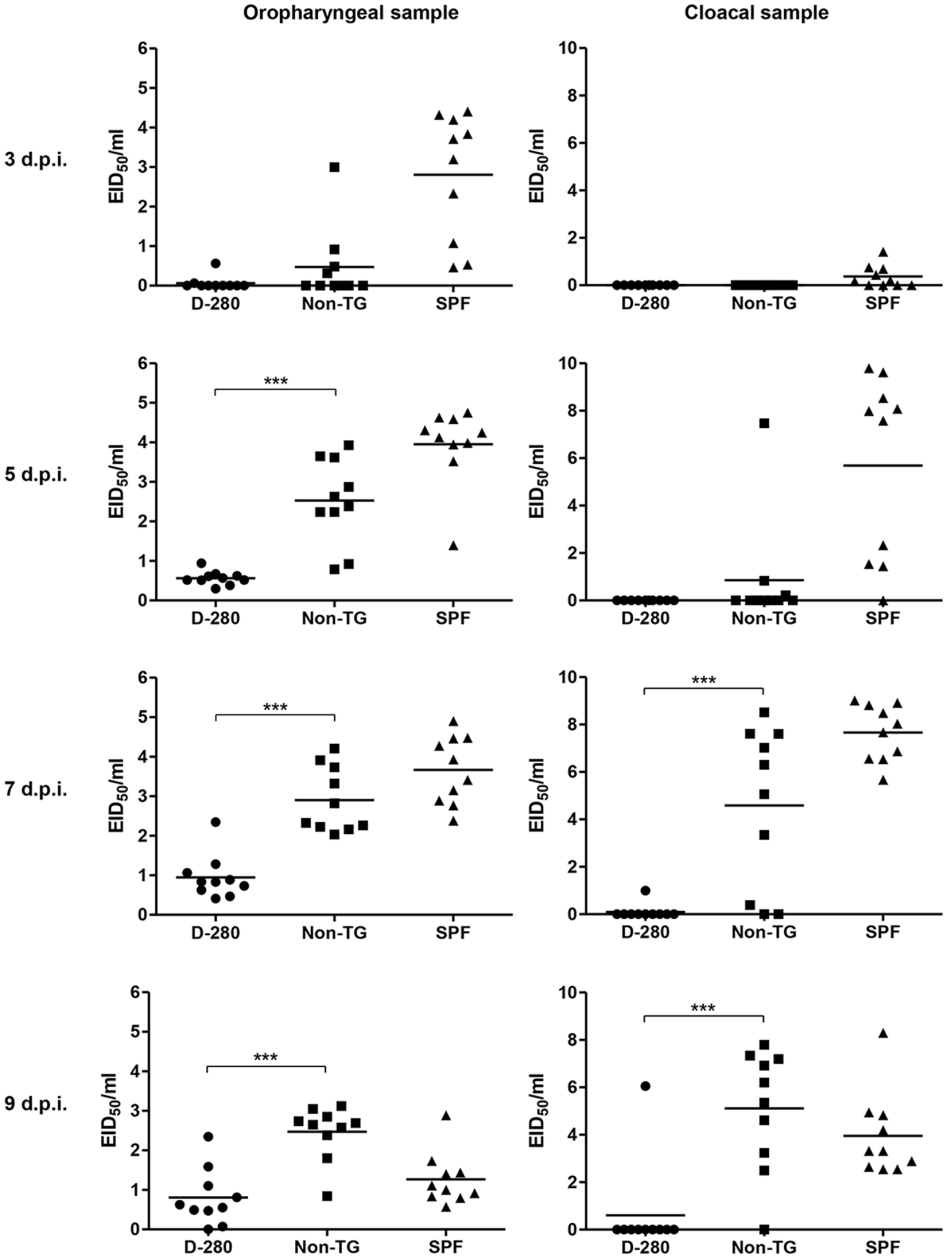
Viral shedding of contact exposed transgenic chickens. Twenty three-week-old non-transgenic chickens (non-TG) and transgenic chickens (D-280) were housed each containment cages in a BSL2 animal facility, and an additional twenty three-week-old SPF chickens were housed as a control group. Ten chickens from each group were intranasally challenged with 100 ${\rm{\mu }}{\rm{l}}$ of 10^7.5^ EID_50_/mL H9N2 virus. The other 10 naïve chickens in each group were co-housed in the same containment isolator from 6 hour post-inoculation. Oropharyngeal and cloacal swab samples were collected at 3, 5, 7, and 9 days post-inoculation (dpi) from contact exposed chickens and the viral RNA was quantified by real-time RT-PCR. Each cycle threshold value was extrapolated to egg infectious dose fifty and mean viral shedding titers were calculated. ***p < 0.001 by unpaired *t*-test.

Transgenic chickens were experimentally infected with AIV to determine whether the expression of the nucleic-acid hydrolyzing antibody conferred reduced viral shedding from the challenged group or reduced susceptibility to infection via contact exposure with the directly infected group. To test whether the H9N2 virus infected the challenged chickens and was successfully transmitted to the contact-exposed chickens under our experimental conditions, SPF chickens were designated as a control group. Direct infection resulted in viral shedding in oropharyngeal samples from all of the directly infected chickens; however, compared with the D-280 and non-TG groups, the SPF group exhibited higher oropharyngeal mean viral shedding titers at 5 and 7 dpi. The cloacal mean viral shedding titers of the D-280 and non-TG groups were also significantly lower than that of the SPF chickens on swabbing days. The D-280 group did not exhibit reduced oropharyngeal viral shedding when directly infected. The only difference observed between the transgenic and non-transgenic chickens was reduced cloacal mean viral shedding titers in the D-280 group at 7 dpi (Fig. 6). To assess the protective capacity of transgenic chickens against contact AIV transmission, viral shedding levels were also measured for contact-exposed transgenic chickens. All of the non-TG and SPF group chickens exhibited oropharyngeal and cloacal viral shedding, suggesting successful contact transmission in our experimental conditions. Interestingly, compared with the direct challenge group, the mean viral shedding titer of the contact-exposed D-280 group was lower than that of non-TG chickens. The D-280 group also showed significantly reduced oropharyngeal viral shedding compared with the non-TG group at 5, 7, and 9 dpi. The non-TG group presented cloacal viral shedding at 7 and 9 dpi, whereas only one D-280 cloacal sample exhibited viral shedding with a significant (p < 0.001) difference in the mean viral shedding titer (Fig. 7).

### Reduced antibody responses to AIV contact exposure in transgenic chickens

**Figure 8:**
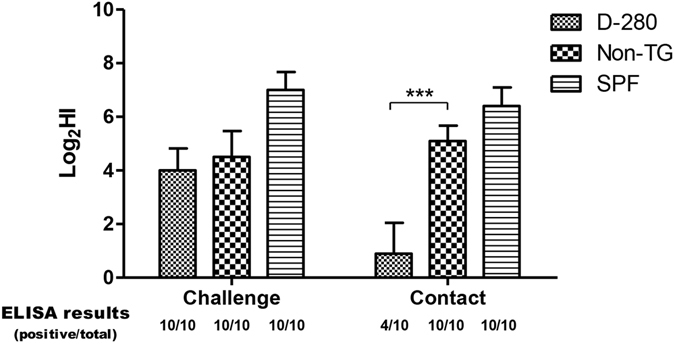
Serum antibody levels and c-ELISA results of challenged and contact exposed chickens. Serum samples were collected three weeks after challenge. Seroconversion was detected using competitive ELISA targeting nucleoprotein antibodies. Mean hemagglutination inhibition (HI) titer were compared between transgenic and non-transgenic chickens. Each error bar represents standard deviation. ***p < 0.001 by unpaired *t*-test.

To identify the evidence of immune response in transgenic chicken following AIV infection, seroconversions were detected using ELISA and HI assays. The ELISA assay detected seroconversion in all of the directly challenged chickens. Seroconversion of all of the contact-exposed non-TG and SPF chickens suggests that the viruses shed by challenged chickens were successfully transmitted; however, contact exposure to AIV induced seroconversion of only four of ten D-280 chickens. The D-280 chickens showed significantly (p < 0.001) reduced mean HI titers (0.9 HIU) than non-TG chickens (5.1 HIU) (Fig. 8). These results indicate that the viruses shed by the D-280 group failed to infect contact-exposed transgenic chickens, whereas the viruses shed by the non-TG group were successfully transmitted.

## Discussion

In this study, we used a recombinant lentiviral vector system to generate transgenic chickens that express the 3D8 single chain variable fragment (3D8 scFv) in all organ tissues. The 3D8 scFv gene encodes a recombinant anti-nucleic acid antibody produced by linking the VH and VL DNA strands of the immunoglobulin gene isolated from an autoimmune-prone MRL-lpr/lpr mouse with lupus disease^17^. Production and characterization of an anti-idiotypic single chain Fv that recognizes an anti-DNA antibody.

Because the 3D8 scFv protein possesses nuclease activity, 3D8 scFv expression is expected to inhibit viral genome amplification regardless of viral shift and drift^21^, and the 3D8 scFv protein exhibited anti-viral activity against classical swine fever virus (CSFV) in PK15 cells^19^. Therefore, 3D8 scFv overexpression in transgenic chickens is predicted to damage the host genome and induce apoptosis because 3D8 scFv exhibits nuclease activity. A study recently demonstrated that 3D8 scFv overexpression in HeLa cells following transient transfection was deleterious and resulted in the degradation of the host nucleic acids^18^. However, low levels of 3D8 scFv expression were tolerated by host cells and did not affect the expression of cytoplasmic RNAs including β-actin mRNA and the 28 S and 18 S rRNAs^19^. Using RT-PCR, we demonstrated that the 3D8 scFv gene is expressed in all transgenic chicken organs, and because 3D8 scFv was expressed at low levels and primarily cytoplasmic, cell death was not induced (confocal figure). Although we did not investigate or verify 3D8 scFv expression levels in fertilized eggs, 3D8 scFv nuclease activity might naturally eliminate embryos overexpressing 3D8 scFv during development.

We characterized the 3D8 scFv gene and protein expression in CEFs derived from 3D8 scFv transgenic chicken (Figs 1, 2 and 3). Although genomic and RT-PCR analyses clearly detected the 3D8 scFv gene in transgenic CEFs, the Western blot analysis of non-immunoprecipitated cell lysates did not detect the 3D8 scFv band. Because confocal microscopy detected a weak green 3D8 scFv signal via a secondary FITC-antibody, we used immunoprecipitation to concentrate the 3D8 scFv proteins in the CEF lysates. The Western blot analysis of the immunoprecipitated lysates revealed an approximately 30 kDa band corresponding to 3D8 scFv protein. Our study demonstrated that the 3D8 scFv gene was consistently expressed in transgenic cells. Because the level of 3D8 scFv expression failed to induce apoptosis, the transgenic cells did not undergo apoptosis. Although we did not intend for the CEFs to express such low levels of 3D8 scFv protein, 3D8 scFv anti-viral activity in transgenic CEFs was detectable. Recently, we have expanded our research towards investigating the role of 3D8 through control of anti-viral effect against H1N1 influenza virus and MNV^22, 23^. Thus, we suppose that low level of 3D8 scFv expression could alleviate initial stage of viral propagation.

The reduction in plaque-forming units suggests that although 3D8 scFv expression levels were lower than expected, viral propagation in the transgenic CEFs was inhibited by the nucleic acid hydrolyzing activity of 3D8 scFv, which resulted in the release of a virus with reduced infectivity towards MDCK cells. Based on our analysis of the anti-viral activity in primary cells derived from transgenic animals, we selected one transgenic chicken lines and examined 3D8 scFv mRNA expression by RT-PCR (Fig. 2). The selected transgenic chicken lines were challenged with a low-pathogenic AI (H9N2). Although AIV subtype H9N2 is categorized as a low pathogenic AI virus that usually replicates in the respiratory and intestinal tracts, it has caused serious economic losses in the Asian poultry industry, including reduced egg production and growth rates. Consequently, we determined whether the transgenic chicken lines could resist the H9N2 virus^24–29^. The expression of the nucleic-acid hydrolyzing antibody in transgenic chickens that were infected with H9N2 virus did not suppress oropharyngeal or cloacal viral shedding; however, although the viral shedding levels were similar in both transgenic and non-TG chickens, virus shed by direct challenge was not enough to infect contact exposed transgenic chickens. Of course, another transmission experiments from AIV infected non-TG chickens to TG chickens are essential to make a better understanding this results. However, when we compared the virus infectivity and virus titers between non-TG chickens and TG chickens by direct infection, the infectivity and virus titers of non-TG and TG chickens were similar whether 3D8 scFv gene is expressed or not. Thus, we could suppose that because viral yields of H9N2 in both group of directly challenged chickens were comparable, so as the overall exposure of the virus in both groups of contact chickens was. The serum antibody results indicated that transgenic chickens that were directly challenged with the virus could not resist systemic H9N2 virus infection; however, as a result of natural transmission, transgenic chickens that were contact exposed to challenged transgenic chickens showed little or no immune response. These results suggest that transgenic chickens that were challenged directly have altered H9N2 virus transmission dynamics because 3D8 scFv weakens viral infectivity. Although the transgenic chicken could not suppress viral shedding from direct infection, they showed some extent of protection from contact transmission. This limited resistance means that the transgenic chicken cannot protect a high dose virus from infection. However, in actual poultry farms, it is suspected that outbreak of AIV starts primarily with virus transmitted by contaminated workers or machineries from outside^30^. In consequence, the virus could infect non-immunized or naïve chickens at first, and spread to adjacent hosts thorough contact or air exposure. We suppose that the transgenic chicken might protect the first exposure of AIV to poultry farm, and also suppress possible chicken-to-chicken and chicken-to-human transmission.

Currently, vaccination is an important tool for controlling AIV infection on poultry farms; however, if the vaccines are not well matched to the strain that the chickens are exposed to, protection conferred by the vaccination can be reduced and the possibility of within-flock transmission may be increased. Consequently, transgenic chickens that can resist AIV could represent a useful built-in resistance concept. To our knowledge, only one trial has been performed using a built-in resistance concept similar to our research^31^. One potential advantage to using the transgenic chickens developed here is that they could reduce continuous within-flock virus transmission by preventing viral replication, thus reducing the likelihood of transmission to people who are in contact with the chickens and ultimately reducing the potential for the spread of AIV from birds to humans. In addition, because we utilized 3D8 scFv that were made to target 18 nucleotide sequence of NP-encoding influenza RNA which is known as relatively conserved sequence regardless of subtype, the 3D8 scFv produced by transgenic chickens has advantage of sequence-specific degradation to influenza virus, which could be applied to other subtypes^32^

In conclusion, we developed a transgenic chicken line expressing 3D8 scFv, and these chickens exhibited an antiviral effect on AIV infection that was confirmed in transgenic chickens and CEFs, thus supporting the idea that 3D8 scFv is an effective anti-viral protein. These results also demonstrate that the transgenic chicken line developed in this study could be useful as a novel strategy for controlling AIV transmission.

## Materials and Methods

### Vector construction and virus particle production

The 0.75-kb 3D8 scFv gene and the 1.7-kb chicken β-actin promoter (CBA) were amplified from pIg20-3D8 scFv^32^ and pCXGFP-1, respectively, using the following primers: 3D8 scFv sense, 5′-ggatccgccaccatggaggtccag-3′ and antisense, 5′-gagctcttttatttccagcttggtccc-3′; CBA sense, 5′-attaatagtaatcaattacggggtc-3′ and antisense, 5′-gaattctttgccaaaatgatgagac-3′. The primers were designed to contain BamHI, XhoI, AseI, or EcoRI sites (underlined) at their 5′ ends. The PCR-amplified fragments were cloned into pCR4-TOPO (Invitrogen, Carlsbad, CA, USA). To introduce the HA-tag sequences into the 3D8 scFv C-terminus, we synthesized oligonucleotides (5′-ctcgagtctagatacccatacgacgtcccagactacgctagctagagggcccgagctcgcggccgc-3′) encoding the HA tag (Bioneer, Korea) and introduced this oligo into the 3D8 scFv/pCR4-TOPO XhoI/NotI sites (Invitrogen). The 3D8 scFv-HA and CBA fragments were digested with BamHI and NotI or AseI and EcoRI, respectively. The digested fragments were then subcloned into the pEGFP-N3 BamHI and NotI or AseI and EcoRI sites, respectively (Clontech, Mountain View, CA, USA). The resulting CBA-3D8 scFv-HA/pEGFP-N3 construct was then digested with AflIII and NotI to generate a 2.5 kb CBA-3D8 scFv-HA fragment that was gel purified and end filled using the Klenow fragment (Promega, Madison, WI, USA). The lentivirus vector DP-IRES-puro (Macrogen, Korea) was digested with HpaI and EcoRV and then ligated to the CBA-3D8 scFv-HA fragment to generate CBA-3D8 scFv-HA-IRES-puro. The CBA-3D8 scFv-HA-IRES-puro DNA sequence was confirmed by sequencing, and a schematic of the construct is shown in Supplementary Figure S1. Lentivirus particles containing the CBA-3D8 scFv-HA gene were produced according to the manufacturer’s instructions (Macrogen). Briefly, three plasmids (the transfer vector, VSV-G expression vector, and gag-pol expression vector) were co-transfected into 293 T cells at a 1:1:1 molar ratio using Lipofectamine 2000 (Invitrogen). Culture media supernatants were then harvested 48 h after transfection, filtered with a 0.45 µm pore membrane filter (Nalgene, NY, USA), and concentrated up to 100 fold using a Centricon Plus-20 centrifugal filter (Millipore, Billerica, MA, USA). Recombinant virus stocks were resuspended in DMEM (Invitrogen), aliquoted, and stored at −80 °C. The concentrated viral stocks contained approximately 1 × 10^8^ colony forming units per milliliter.

### Generation of transgenic chickens

The embryos were cultured until stage-X and microinjected with lentivirus vector as previously described^33^. Approximately 100,000 virus particles were microinjected into the center of the subgerminal cavity below the developing embryo of each newly laid egg produced by Lohmann brown laying hens. The embryos were incubated until hatching using the surrogate shell *ex vivo* culture system phases II and III, and the chicks that hatched were raised to sexual maturity. To screen the transgenic roosters for the presence of the transgene, genomic DNA was extracted from the semen of hatched roosters (G0) using the Wizard® Genomic DNA Purification Kit (Promega). A PCR assay (50 μl volume) was performed using FastStart PCR master (Roche Applied Science, Indianapolis, IN, USA), 50 ng of genomic DNA template, and 100 nM of each primer (sense, 5′-cctctgctaaccatgttcatgccttc-3′ and antisense, 5′-gctagtgaatgtgtatccagaagcctt-3′) to amplify 260 bp DNA fragments, including the β-actin promoter and 3D8 scFv gene. The PCR assay was performed under the following conditions: initial DNA denaturation at 95 °C for 4 min; 35 sets of incubations at 95 °C for 30 s, 60 °C for 30 s, and 72 °C for 20 s; and a final incubation at 72 °C for 5 min. The transgenic roosters were then mated with wild-type hens to produce G1 and G2 transgenic chickens. Transgene insertions in individual G1 and G2 chickens were analyzed first by PCR and subsequently by Southern blot analysis. Genomic DNA (10 μg) was isolated from whole blood, digested with EcoRV (Promega), resolved on a 1.0% (w/v) agarose gel, and then transferred to a nylon membrane (Roche). EcoRV cuts once within the integrated provirus between the HA tag and the IRES (see Supplementary Figure S1). The membrane was analyzed with DIG-labeled 3D8 scFv probes (25 ng/ml) using the DIG High Prime DNA Labeling and Detection Starter Kit II (Roche).

### 3D8 scFv gene expression in transgenic chickens

To analyze the 3D8 scFv mRNA expression patterns in transgenic chickens, tissue samples were harvested and homogenized using TRIzol and a PureLink® RNA Mini Kit (Invitrogen) according to the manufacturer’s protocol. Total RNA was isolated from the chicken embryonic fibroblasts (CEFs) and organ tissues (e.g., trachea, ovary/testis, kidney, spleen, pancreas, brain, lung, liver, heart, muscle, and intestine) of an adult G2 hemizygous transgenic and a wild-type hen. cDNA was synthesized from 2 μg of total RNA using the ImProm-IITM reverse transcription system (Promega) according to the manufacturer’s instructions. One microliter of cDNA was used for each 50 μl PCR reaction, which were performed using Ex Taq DNA polymerase (TaKaRa, Tokyo, Japan). The following primers were used for the PCR amplifications: 3D8 scFv sense, 5′-actctcaccatcagcagtgtgcag-3′ and antisense, 5′-ctagctagcgtagtctgggacg-3′; chicken GAPDH sense, 5′-gattctacacacggacacttcaagg-3′ and antisense, 5′-acaatgccaaagttgtcatggatgac-3′^34^. Following an initial cDNA denaturation at 95 °C for 2 min, the PCR amplification conditions consisted of denaturation at 95 °C for 20 s, annealing at 60 °C for 40 s, and extension at 72 °C for 30 s. Thirty-five amplification cycles were performed for 3D8 scFv and 25 cycles were performed for GAPDH. The PCR products were resolved on 1.5% agarose gels, and GAPDH amplification was used to normalize cDNA loading.

### 3D8 scFv transgenic chicken embryonic fibroblast cells

To prepare the primary CEFs, the body walls of 10-day-old chicken embryos were chopped with a knife, trypsinized, and collected by centrifugation^35^. Enzymatic activities were stopped with fetal bovine serum (FBS), and the cells were washed once with PBS. The culture media contained 15% FBS (HyClone, Logan, UT), 100 U/ml penicillin (HyClone), and 100 µg/ml streptomycin (HyClone). The harvested cells were plated and maintained at 37 °C with 5% CO_2_. The 3D8 scFv transgenic cells isolated from transgenic eggs were selected by 2 µg/ml Puromycin (Sigma, St Louis, MO) for 3 days. The surviving cells were selected and amplified in the same media containing 2 µg/ml Puromycin for subsequent experiments. The amplified cells were frozen in Recovery™ Cell Culture Freezing Media (Gibco, Grand Island, NY, USA) for later experiments.

### Western blot analysis

Western blot analysis was performed to assess 3D8 scFv protein expression in the transgenic CEFs (D-280, offspring of P-54; E-185, offspring of N-165). Cell lysates were produced using wild-type and transgenic CEFs and then immunoprecipitated with protein G-agarose beads (Roche) and 5 µl of polyclonal rabbit anti-HA tag antibodies (Abcam, Cambridge, UK) at 4 °C overnight. The precipitated proteins were resolved by SDS-PAGE and transferred to a nitrocellulose membrane (Roche). The membrane was then immersed in blocking solution containing 5% skim milk in TBS-T at room temperature for 1 h and incubated with 1 µg/ml of a monoclonal mouse anti-HA tag antibody (Abcam) in TBS-T at 4 °C overnight. The membrane was then washed 3 times for 10 min with TBS-T and incubated with the appropriate HRP-conjugated second antibody in 5 TBS-T with % skim milk at room temperature for 1 h. The blot was then washed 5 times with TBS-T, and the specific antibody-antigen complexes were detected using an ECL detection system (GE Healthcare, Fairfield, CT, USA).

### Confocal laser scanning microscopy and immunofluorescence analysis

Transgenic (D-280) and wild-type CEFs were seeded at a density of 5 × 10^4^ cells/well in a 4-well plate over glass coverslips the day before use. The cells were fixed with 4% paraformaldehyde for 15 min, washed 3 times with cold PBS 3 times and then permeabilized with Perm buffer (1% BSA, 0.1% saponin, and 0.1% sodium azide in PBS) at room temperature for 10 min. The cells were blocked with 3% horse serum in PBS for 40 min and then incubated with rabbit anti-HA tag Ab followed by FITC-anti-rabbit IgG (Invitrogen). The nuclei were stained by incubating cover slips with 100 µg/ml propidium iodide at room temperature for 5 min. The cells on the coverslips were mounted in mounting media, observed using a Zeiss LSM 510 laser confocal microscope and analyzed with Carl Zeiss LSM Image software.

### CEF viral infection

CEFs from two G2 hemizygous transgenic progeny (D-280 and E-185) and non-transgenic embryos were prepared and cultured in M199 (Invitrogen, Germany) and Ham’s F-10 (Sigma, St Louis, MO) mixed media containing 10% FBS (Hyclone, Logan, UT, USA), 0.65% sodium bicarbonate, and 1% penicillin/streptomycin. The CEFs were prepared 24 h prior to H9N2 infection at 10^7.5^ EID_50_/200 µl, and the infections were performed in triplicate. After 48 h, confluent MDCK monolayers that had been propagated in 6-well plates were infected with an undiluted supernatant and a 10-fold diluted supernatant from the H9N2 virus-infected CEFs. After infection, the cells were washed with PBS and overlaid with a 0.7% agarose gel (Invitrogen) containing 1 ${\rm{\mu }}{\rm{g}}$/ml tosylsulfonyl-phenylalanyl-chloromethyl ketone (TPCK)-treated trypsin (Sigma). After 48 h, the cells were fixed in 10% PBS-buffered formalin and plaques were visualized using crystal violet staining.

### *In vivo* viral infection and transmission studies

Twenty three-week-old non-transgenic chickens (non-TG) and transgenic chickens (D-280) were housed in containment cages in a BSL2 animal facility, and an additional twenty three-week-old SPF chickens were housed as a control group. All chickens were identified using individual tags, and feed and water were supplied *ad libitum*. To confirm that the chickens were serologically naïve to AI, we utilized a commercially available competitive ELISA (BioNote, Korea) directed against anti-nucleoprotein (NP) antibodies. Ten chickens from each group were intranasally challenged with 100 ${\rm{\mu }}{\rm{l}}$ of 10^7.5^ EID_50_/mL H9N2 virus (A/Korean native chicken/Korea/K040110/2010). Ten naïve chickens in each group were co-housed in the same containment isolator for 6 hours post-inoculation. To assess viral shedding, oropharyngeal and cloacal swab samples were collected at 3, 5, 7, and 9 days post-inoculation (dpi) and suspended in 1 mL of PBS. Suspensions (200 ${\rm{\mu }}{\rm{l}}$) were used for RNA extraction using an RNeasy Mini Kit (QIAGEN, Valencia, CA, USA) according to the manufacturer’s instructions. Viral RNA was quantified by cycle threshold (Ct) values using M gene-based real-time RT-PCR as previously described^36^. To extrapolate the Ct values to infectious units, serial 10-fold dilutions were prepared from known titers of A/Korean native chicken/Korea/K040110/2010 (H9N2) viruses from egg allantoic fluid (measured in EID_50_). Viral RNA was extracted from these dilutions and quantified by real-time RT-PCR, as described above. To generate a standard curve, the Ct values for each viral dilution were plotted against the viral titers. The resulting standard curve had a high correlation coefficient (r^2^ > 0.99) and was used to determine the EID_50_ from Ct values. To detect the immune response to challenged virus, serum samples were collected three weeks after challenge for hemagglutination inhibition (HI) assays. HI assays were performed according to the OIE standard HI method using 4 hemagglutination units (HAU) of formalin-inactivated homologous antigen^37^.

### Ethics statements

The generation of transgenic chickens was reviewed and approved by the Korea National Institute of Animal Science Institutional Animal Care and Use Committee (IACUC) (2013–051). The experimental procedures used in animal management, reproduction, and embryo manipulation followed the standard operating procedures of our laboratory. Transgenic chicken infections were performed at the Konkuk University School of Veterinary Medicine and approved by the Konkuk University IACUC.

### Statistical analysis

An analysis of variance (ANOVA) and Tukey–Kramer post-hoc test were used to compare the plaque reduction percentages. Unpaired t-tests were used to compare viral shedding and serum HI titers between transgenic chickens and non-transgenic chickens. P-values < 0.05 were considered to be significant.

## Electronic supplementary material

Supplementary Datasets

## References

[1] Herfst S, Imai M, Kawaoka Y, Fouchier RA (2014). Avian influenza virus transmission to mammals. Current topics in microbiology and immunology.

[2] Capua I, Marangon S (2007). Control and prevention of avian influenza in an evolving scenario. Vaccine.

[3] Hensley SE (2009). Hemagglutinin receptor binding avidity drives influenza A virus antigenic drift. Science.

[4] Mumford JA (2007). Vaccines and viral antigenic diversity. Revue scientifique et technique.

[5] Lee DH (2011). Antiviral efficacy of oseltamivir against avian influenza virus in avian species. Avian diseases.

[6] Lee HJ (2012). Anti-influenza virus activity of green tea by-products *in vitro* and efficacy against influenza virus infection in chickens. Poultry science.

[7] Medina RA, Garcia-Sastre A (2011). Influenza A viruses: new research developments. Nature reviews. Microbiology.

[8] Li YC, Kong LH, Cheng BZ, Li KS (2005). Construction of influenza virus siRNA expression vectors and their inhibitory effects on multiplication of influenza virus. Avian diseases.

[9] Stewart CR, Karpala AJ, Lowther S, Lowenthal JW, Bean AG (2011). Immunostimulatory motifs enhance antiviral siRNAs targeting highly pathogenic avian influenza H5N1. PloS one.

[10] Meng S (2011). Recombinant chicken interferon-alpha inhibits H9N2 avian influenza virus replication *in vivo* by oral administration. Journal of interferon & cytokine research: the official journal of the International Society for Interferon and Cytokine Research.

[11] Stewart CR (2012). Toll-like receptor 7 ligands inhibit influenza A infection in chickens. Journal of interferon & cytokine research: the official journal of the International Society for Interferon and Cytokine Research.

[12] Barjesteh N, Brisbin JT, Behboudi S, Nagy E, Sharif S (2015). Induction of antiviral responses against avian influenza virus in embryonated chicken eggs with toll-like receptor ligands. Viral immunology.

[13] Seo BJ (2012). Evaluation of Leuconostoc mesenteroides YML003 as a probiotic against low-pathogenic avian influenza (H9N2) virus in chickens. Journal of applied microbiology.

[14] Saxena SK (1996). Inhibition of HIV-1 production and selective degradation of viral RNA by an amphibian ribonuclease. The Journal of biological chemistry.

[15] Youle RJ (1994). RNase inhibition of human immunodeficiency virus infection of H9 cells. Proceedings of the National Academy of Sciences of the United States of America.

[16] Sano T, Nagayama A, Ogawa T, Ishida I, Okada Y (1997). Transgenic potato expressing a double-stranded RNA-specific ribonuclease is resistant to potato spindle tuber viroid. Nature biotechnology.

[17] Kwon MH (2002). Production and characterization of an anti-idiotypic single chain Fv that recognizes an anti-DNA antibody. Immunological investigations.

[18] Jang JY (2009). A nucleic acid-hydrolyzing antibody penetrates into cells via caveolae-mediated endocytosis, localizes in the cytosol and exhibits cytotoxicity. Cellular and molecular life sciences: CMLS.

[19] Jun HR (2010). An RNA-hydrolyzing recombinant antibody exhibits an antiviral activity against classical swine fever virus. Biochemical and biophysical research communications.

[20] Lee G (2014). A nucleic-acid hydrolyzing single chain antibody confers resistance to DNA virus infection in hela cells and C57BL/6 mice. PLoS pathogens.

[21] Kim YR (2006). Heavy and light chain variable single domains of an anti-DNA binding antibody hydrolyze both double- and single-stranded DNAs without sequence specificity. The Journal of biological chemistry.

[22] Cho S (2015). Preventive Activity against Influenza (H1N1) Virus by Intranasally Delivered RNA-Hydrolyzing Antibody in Respiratory Epithelial Cells of Mice. Viruses.

[23] Hoang PM (2015). Development of Lactobacillus paracasei harboring nucleic acid-hydrolyzing 3D8 scFv as a preventive probiotic against murine norovirus infection. Applied microbiology and biotechnology.

[24] Perk S (2009). Phylogenetic analysis of hemagglutinin, neuraminidase, and nucleoprotein genes of H9N2 avian influenza viruses isolated in Israel during the 2000-2005 epizootic. Comparative immunology, microbiology and infectious diseases.

[25] Choi YK (2004). Continuing evolution of H9N2 influenza viruses in Southeastern China. Journal of virology.

[26] Guo YJ (2000). Characterization of the pathogenicity of members of the newly established H9N2 influenza virus lineages in Asia. Virology.

[27] Lee YJ (2007). Continuing evolution of H9 influenza viruses in Korean poultry. Virology.

[28] Naeem K, Ullah A, Manvell RJ, Alexander DJ (1999). Avian influenza A subtype H9N2 in poultry in Pakistan. The Veterinary record.

[29] Lee YN (2011). Isolation and characterization of a novel H9N2 influenza virus in Korean native chicken farm. Avian diseases.

[30] FAO. in *FAO ANIMAL PRODUCTION AND HEALTH PAPER* (Food and Agricultrue Organization of the United Nations, Rome, Italy, 2008).

[31] Lyall J (2011). Suppression of avian influenza transmission in genetically modified chickens. Science.

[32] Kim A, Lee JY, Byun SJ, Kwon MH, Kim YS (2012). Viral genome RNA degradation by sequence-selective, nucleic-acid hydrolyzing antibody inhibits the replication of influenza H9N2 virus without significant cytotoxicity to host cells. Antiviral research.

[33] Byun, S. J. *et al*. Oviduct-specific enhanced green fluorescent protein expression in transgenic chickens. *Biosci Biotechnol Biochem* **75**, 646–649, doi:JST.JSTAGE/bbb/100721 [pii] (2011). 10.1271/bbb.100721 21512248

[34] Thiels E (2000). Impairment of long-term potentiation and associative memory in mice that overexpress extracellular superoxide dismutase. J Neurosci.

[35] Maas R, van Zoelen D, Oei H, Claassen I (2006). Replacement of primary chicken embryonic fibroblasts (CEF) by the DF-1 cell line for detection of avian leucosis viruses. Biologicals: journal of the International Association of Biological Standardization.

[36] Spackman E (2003). Development of real-time RT-PCR for the detection of avian influenza virus. Avian diseases.

[37] Swayne, D. & Brown, I. *Manual of Diagnostic Tests and Vaccines for Terrestrial Animal*, *Chapter 2.3.4. Avian Influenza*, http://www.oie.int/fileadmin/Home/eng/Health_standards/tahm/2.03.04_AI.pdf (2015).

