# Supplementary material for: Transgenic Chickens Expressing the 3D8 Single Chain Variable Fragment Protein Suppress Avian Influenza Transmission
Source: Sci Rep. 2017 Jul 19;7:5938. doi: 10.1038/s41598-017-05270-8 (PMC5517518; doi:10.1038/s41598-017-05270-8)
Supplement: Supplementary file 1 — Supplementary Datasets [file 41598_2017_5270_MOESM1_ESM.doc]

**Transgenic Chickens Expressing the 3D8 Single Chain Variable Fragment Protein Suppress Avian Influenza Transmission**

Sung June Byun^1,3^, Seong-su Yuk^2,3^, Ye-Jin Jang^1^, Hoonsung Choi^1^, Mi-Hyang Jeon^1^, Erdene-Ochir TO^2^, Jung-Hoon Kwon^2^, Jin-Yong Noh^2^, Jeom Sun Kim^1^, Jae Gyu YOO^1^, Chang-Seon Song^2†^

^1^Animal Biotechnology Division, National Institute of Animal Science, Rural Development Administration, Suwon, 441-706, Republic of Korea

^2^ Department of Avian Disease Laboratory, College of Veterinary Medicine, Konkuk University, Seoul, Korea

^3^ These authors contributed equally to this work.


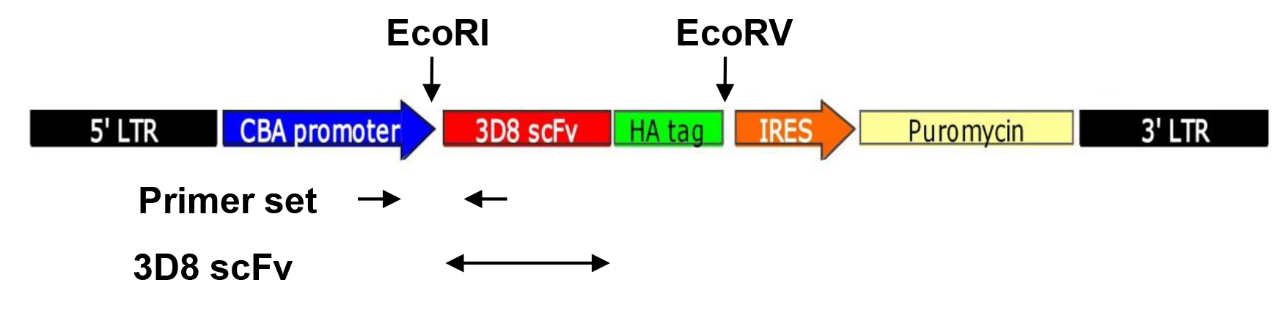


Supplementary Figure S1. Structure of the pLenti-CBA-3D8 scFv-HA-IRES-puro vector. LTR, long terminal repeat; CBA, chicken β-actin promoter; HA, HA-tag; IRES, internal ribosome entry site; puro, puromycin resistance gene.

Supplementary Table S2. PCR screening for the 3D8 scFv gene in G0 rooster semen.

| Rooster carrying the 3D8 scFv gene in their semen | Number of G1 chicks PCR screened for the 3D8 scFv | G1 chicks carrying the 3D8 scFv in their blood |
| --- | --- | --- |
| J-21 | 459 | 6 (N-82, N-95, N-140, N-165, N-183, and N-238) |
| J-168 | 335 | - |
| J-186 | 508 | 3 (P-54, P-196, and P-296) |


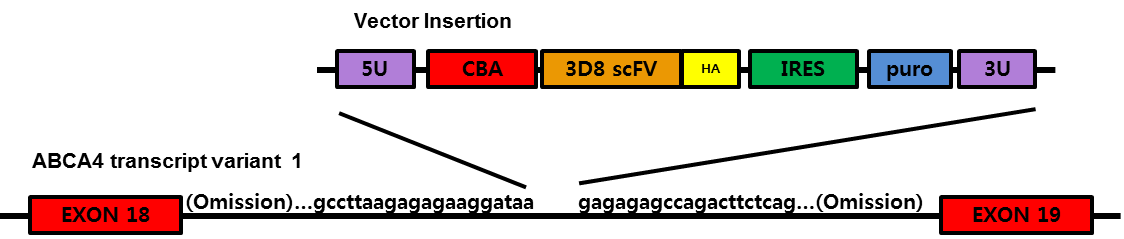


Supplementary figure S3. The vector was inserted into the intron sequence between exon 18 and 19 of ABCA4 transcript variant 1.

Supplementary figure S4. Comparison of body weight between 3D8 scFv transgenic chicken and other transgenic chickens. Each of the ten body weights was measured at 30 weeks age (ten chickens in each group). All transgenic chickens were generated using same method as 3D8 scFv transgenic chicken. Wild-type means body weight of Lohman brown laying hens. GFP, Green fluorescent protein; GR2 GFP, Glyoxylate reductase green fluorescent protein; SOD3, Superoxide dismutase; LTF, Lactotransferrin; EPO, Erythropoietin; Tet-GFP, Tetracyclin repressor and green fluorescent protein.


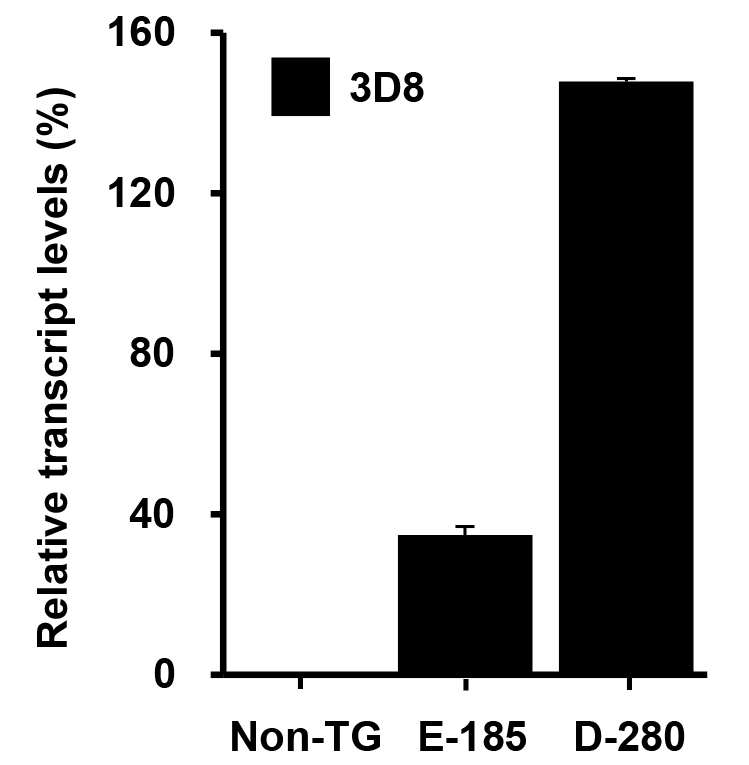


Supplementary figure S5. RT-PCR analysis of 3D8 scFv mRNA in bronchi of transgenic and wild-type chicken. The trachea of 3D8 scFv transgenic (E-185 and D-280) and non-transgenic (Non-TG) were subjected to quantitative polymerase chain reaction (qPCR) analyses of 3D8 signal. Relative transcript and protein levels were normalized to the expression of glyceraldehyde phosphate dehydrogenase (GAPDH).
